# PTPN12 down-regulated by miR-146b-3p gene affects the malignant progression of laryngeal squamous cell carcinoma

**DOI:** 10.1515/med-2023-0727

**Published:** 2023-06-09

**Authors:** Lili Lan, Huan Cao, Lei Zhao, Weina Cui, Baoshan Wang

**Affiliations:** Otolaryngology Head and Neck Surgery Department, The Second Hospital of Hebei Medical University, Shijiazhuang 050005, Hebei, China; Otolaryngology Head and Neck Surgery Department, The Fourth Hospital of Hebei Medical University, Shijiazhuang 050011, Hebei, China; Otolaryngology Head and Neck Surgery Department, The Second Hospital of Hebei Medical University, 215 Heping West Road, Shijiazhuang 050005, Hebei, China

**Keywords:** PTPN12, Laryngeal squamous cell carcinoma, migration, invasion, miR-146b-3p

## Abstract

Laryngeal squamous cell carcinoma (LSCC) is a common malignancy among men in the anatomical position of head and neck. Hoarseness, pharyngalgia, and dyspnea are common symptoms. LSCC is a complex polygenic carcinoma that is caused by many factors involving polygenic alteration, environmental pollution, tobacco, and human papillomavirus. Classical protein tyrosine phosphatase nonreceptor type 12 (PTPN12) has been extensively studied to decipher its mechanism as a tumor suppressor gene in various human carcinomas; however, there is no comprehensive elucidation of the PTPN12 expression and its regulatory mechanisms in LSCC. As such, we expect to provide new insights for finding new biomarkers and effective therapeutic targets in LSCC. Immunohistochemical staining, western blot (WB), and quantitative real-time RT-PCR (qRT-PCR) were used for the messenger RNA (mRNA) and protein expression analyses of PTPN12, respectively. 3-(4,5-dimethylthiazol-2-yl)-5-(3-carboxymethoxyphenyl)-2-(4-sulfophenyl)-2*H*-tetrazolium, clone formation, transwell migration, and transwell invasion assays were used to assess the proliferation, migration, and invasion ability of LSCC cells. Online prediction and design software tools (http://www.targetscan.org/ and http://www.microRNA.org) were used to predict associated miRNA. Studying the targeted regulatory relationship between miR-146b-3p and PTPN12 was based on dual luciferase reporter gene analysis. qRT-PCR was used to assess miR-146b-3p expression in LSCC. miR-146b-3p inhibitor and mimic were transfected, followed by qRT-PCR and WB assays to detect the expression of PTPN12. The gain and loss functional experiments were used to investigate the effects of miR-146b-3p transfection on the proliferation, migration, and invasion of tumor cells. Online bioinformatics prediction software (https://cn.string-db.org/ and https://www.genecards.org/) was used to determine potential downstream target genes of PTPN12. qRT-PCR and WB analyses were used to assess the mRNA and protein expression levels of target genes. Our study showed significantly decreased mRNA and protein expression levels of PTPN12 in LSCC compared with the adjacent normal tissues. The lower PTPN12 mRNA expression was correlated with pathological differentiation, and lower PTPN12 protein expression was correlated with the TNM stage in LSCC tissues. The subsequent *in vitro* functional analyses showed the inhibitory effect of PTPN12 over-expression on the proliferation, migration, and invasiveness abilities of LSCC cell line. Using online prediction and design software, miR-146b-3p was searched to target PTPN12. The miR-146b-3p was expressed at a high level in LSCC tissues and cell lines. Luciferase reporter assay exhibited that miR-146b-3p inhibited the luciferase activity of PTPN12 markedly. The functional analyses showed the tumor-promoting role of miR-146b-3p on the proliferation, migration, and invasiveness abilities of LSCC cell. Furthermore, co-transfection of cells with miR-146b-3p and PTPN12 significantly restored the inhibitory effect of PTPN12 on LSCC cell growth, migration, and invasiveness. This phenomenon unveiled that miR-146b-3p regulated the proliferation, migration, and invasion of LSCC cells by targeting PTPN12. EGFR and ERBB2 were selected as the downstream-regulation target genes. Up-regulation of PTPN12 significantly suppressed EGFR expression. Accordingly, the miR-146b-3p mimic significantly up-regulated the EGFR expression. However, up-regulation of PTPN12 and miR-146b-3p mimic suppressed ERBB2 protein expression but induced its gene expression. Down-regulation of PTPN12 is associated with up-regulation of miR-146b-3p in LSCC. Moreover, PTPN12 serves as a tumor suppressor gene through regulating the proliferation, migration, and invasion of LSCC cells. miR-146b-3p/PTPN12 axis is expected to be a novel therapeutic target in LSCC.

## 1 Introduction

Laryngeal squamous cell carcinoma (LSCC) is one of the most common malignancies of head and neck, ranked as the 11th most common tumor among male malignancies and 2nd among all respiratory malignancies [1]. With teenage smoking and tobacco consumption increasing, the incidence of LSCC has risen and displayed a trend of younger age than past few decades. There were 184,615 new cases and 99,840 deaths worldwide in 2020 from the World Health Organization, accounting for 1% of all cancers diagnosed and 1% of cancer deaths, respectively [2]. Postoperative recurrence and metastasis were the main causes of treatment failure or death in LSCC patients [3]. To find valuable molecular markers for high-risk individuals screening, risk assessment and prognosis judgment is an effective intervention to reduce the incidence and mortality of LSCC.

Protein phosphorylation is one of the most common post-transcriptional modifications participating in signal transduction, cell regulation, protein interaction, gene transcription, immune response, and metabolism [4,5]. Protein tyrosine phosphatases (PTPs) regulate the equilibrium of tyrosine phosphorylation and protein tyrosine phosphokinases (PTKs), meanwhile, they serve as tumor inhibitors in many malignancies. This equilibrium is crucial for maintaining the immune system, conversely, disruption to the balance can produce immune deficiency diseases, autoimmune diseases, or malignant tumors [6]. PTP non-receptor Type 12 (PTPN12) is a novel tumor suppressor gene participating in many malignancies’ occurrence and development [7]. PTPN12 was first found in intestinal tissues and located on chromosome 7q11.23. PTPN12 plays an important role in antitumor and cell physiology [5]. Abnormal activation of PTKs and absence of PTPN12 have been observed in breast cancer, lung cancer, and brain malignancies [8,9,10]. In recent years, PTPN12 has attracted much attention due to its role in tumor suppression by restraining PTKs. However, there is no comprehensive elucidation of the PTPN12 expression, regulatory mechanism, and its role in LSCC occurrence and development.

microRNAs (miRNAs) generated by RNA polymerase II (Pol II), are a class of endogenous single-stranded non-coding RNAs with a length of 19–25 nucleotides. In the last decade, miRNAs changed to be a favored topic for research due to their diverse functionality. miRNAs play regulating roles in almost all aspects of solid tumor biology, such as cell proliferation, invasion, adherence, metastasis, and apoptosis[11]. In addition to these, aberrantly expressed miRNAs were detected in different tissues, distinct tumor types, and tumor stages. Therefore, some miRNAs have been reported to serve as biomarkers of diagnosis and prognosis for malignant tumors, which are becoming vital therapeutic targets for cancer, and provide new insights for tumor treatment [12]. miRNAs can also interact with long non-coding RNAs (lncRNAs) to influence miRNA-induced targeted gene expression, for instance, let-7c-5p interacts with TMPO-AS1 to the up-regulates targeted gene STRIP2 expression [13]. miRNAs post-transcriptionally repress the target messenger RNA (mRNA) expression by binding of the 3′-untranslational region (3′-UTR) of target mRNA, eventually leading to mRNA degradation or gene silencing. Recent accumulating evidence has uncovered that abnormal expressed miRNAs play essential roles in many kinds of tumors, such as breast cancer [14], retinoblastoma [15], and prostate cancer [16]. However, to date, molecular mechanisms by which miRNA-mediated PTPN12 affects LSCC initiation and progression are poorly elaborated.

In our study, we conducted research to investigate the expression and the role of PTPN12 in the development of LSCC for the first time. We found that PTPN12 expression was decreased in LSCC tissues and cell lines and exerted an inhibitory effect of PTPN12 over-expression on proliferation, migration, and invasiveness abilities of LSCC cell lines. Subsequently, we searched for the associated miRNA of PTPN12 and further explored its functions in the progression of LSCC. miR-146b-3p, which directly targets PTPN12, was up-regulated in LSCC tissues, contributing to increased LSCC cell proliferation, migration, and invasion. Our findings collectively suggest that PTPN12 is one of the target genes of miR-146b-3p. In addition, the downstream target genes were predicted in an attempt to understand the underlying mechanism. Our studies revealed, for the first time, the mechanism by which miR-146b-3p regulates PTPN12 expression in LSCC.

## 2 Materials and methods

### 2.1 Clinical samples collection

LSCC tissues and corresponding adjacent normal tissues were obtained from 60 surgical patients without any antitumor therapy between 2016 and 2020 in Biobank of Otorhinolaryngology Head and Neck Surgery of Hebei Medical University (Shijiazhuang, China). All specimens were obtained with signed informed consent. All experimental approaches performed were according to the Helsinki Declaration (as revised in 2013) and ethical standards. Our study was approved by the Ethics Committee of the Fourth Hospital of Hebei Medical University (Ethical Approval Number: 2021KY234). Tissues were divided into two parts, one part was used for genomic RNA extraction and the rest part was fixed with 4% neutral formaldehyde solution for preparation of paraffin sections. According to the American Joint Committee on Cancer 8th edition staging criteria, patients were structured into stages I, II, III, and IV [17]. Smokers were defined as former or current individuals smoking five cigarettes per day for at least 2 years [18]. The patients were all male, with a median age of 62 years (47–69 years). Patients’ clinical characteristics were detailed in Table 1.

**Table 1:** Expression of PTPN12 mRNA in LSCC and its relationship with clinicopathological features

Characteristics	*N*	Expression level of PTPN12 mRNA	
		*Z*	*P*
Age (years)			
<60	21	−1.839	0.066
≥60	39		
Smoking			
No	6	−0.034	0.973
Yes	54		
TNM stage			
Ⅰ + Ⅱ	29	−1.005	0.315
Ⅲ + Ⅳ	31		
Lymph node metastasis			
N0	36	−1.430	0.153
N1/2/3	24		
Pathological differentiation			
Well/moderate	51	−1.990	0.047
Poor	9		

### 2.2 LSCC cell culture

Human LSCC cell lines TU177 and AMC-HN-8 were purchased from BNBIO (Beijing, China), stored, and subcultured in the Biobank of Otolaryngology Head and Neck Surgery of Hebei Medical University. TU177 cells were cultured in a RPMI-1640 medium (GIBCO, NY, USA), whereas AMC-HN-8 cells were grown in Dulbecco’s modified Eagle’s medium (Invitrogen, CA, USA). Both were supplemented with 10% fetal bovine serum (FBS; Invitrogen) in an incubator. The condition of the incubator was maintained at 37°C with 5% CO_2_.

### 2.3 Extraction of total RNA and quantitative real-time RT-PCR (qRT-PCR)

Total RNA was extracted from LSCC cells, LSCC tissues, and paired corresponding adjacent normal tissues according to the instructions and reversed transcribed to cDNA using the Transcriptor First Strand cDNA Synthesis Kit (Roche, Vaud, Switzerland). Synthetic cDNA was used as template and GAPDH was used as internal reference for qRT-PCR amplification. Primers are listed in Table A1. The qRT-PCR conditions were as follows: pre-denaturation at 95°C for 10 min, denaturation at 95°C for 15 s, annealing at 59°C for 30 s, extension at 72°C for 30 s, a total of 35 cycles, and extension at 72°C for 7 min. The relative expression of PTPN12 was quantified by the 2^−△△Ct^ [19]. The experiments were assayed in triplicate.

### 2.4 Immunohistochemical (IHC) staining

The paraffin sections were dewaxed, dehydrated, repaired with EDTA Buffer (PH = 9.0) continued for 2.5 min, and inactivated endogenous peroxidase. The primary anti-PTPN12 antibody was added (Abcam, Cambridge, UK, dilution 1:50) to sections and then incubated at 37°C for 3 h. Biotinylated second antibody and horseradish peroxidase-labeled working solution were dropped sequentially to the sections. DAB reagent was used for chromogen detection. Negative control groups were dropped with PBS instead of primary antibody. IHC staining results were evaluated and scored by two experienced pathologists in a double-blind way. The scores were calculated according to the percentage of positive cells and the staining intensity. The cells that present yellow or brown material in the cytoplasm were defined as positive cells. In our experiment, the total IHC scores were determined by summing each staining intensity and extent. Scores 0–2 were considered negative-expression, and scores 3–6 were positive.

### 2.5 Western blotting (WB)

PTPN12 protein expression differences were analyzed by WB in LSCC cells and normal tissues. LSCC cell lines (AMC-HN-8 and TU177) and five cases of normal tissue protein extracts were lysed using a radioimmunoprecipitation assay lysis buffer (Elabscience Biotechnology Co., Ltd., Wuhan, China) and subjected to ultrasonic shaking. Each sample was adjusted for equal amounts and masses and fractionated using 10% SDS-polyacrylamide gel electrophoresis. The transferred membranes were blocked with 5% milk for 1 h and probed with anti-PTPN12 antibodies (Abcam, Cambridge, UK, dilution 1:100). The bands were visualized using ECL (Sangon Biotech Co., Ltd., Shanghai, China), and an anti-β-actin antibody (Abcam, Cambridge, UK) was used as a control.

### 2.6 PTPN12 over-expression vector construction and transfection

The PTPN12 over-expression vector pcDNA3.1-PTPN12 and an empty pcDNA3.1 vector pcDNA3.1-NC were constructed by Sangon Biotech Co., Ltd. (Shanghai, China). Cells were evenly seeded in a six-well plate after digestion and cell counting. Transfection treatment was performed when cells were 70–80% confluent. The two plasmid transfections were performed using Lipofectamine 2000 (Invitrogen, Beijing, China) according to manufacturer instructions. qRT-PCR and WB analyses were carried out to calculate transfection efficiency 24 h post-transfection.

### 2.7 MTS and clone formation assays

Cell proliferation activity of LSCC cells with PTPN12 over-expression was measured by 3-(4,5-dimethylthiazol-2-yl)-5-(3-carboxymethoxyphenyl)-2-(4-sulfophenyl)-2*H*-tetrazolium (MTS) and clone formation assays *in vitro*. MTS assay was performed as follows: the cells with a cell concentration of 2,000 cells/100 μl each well were seeded into 96-well plates, and six replicate holes were designed for each group. The absorbance optical density values were evaluated at 490 nm in 0, 24, 48, 72, and 96 h after cell adherence. Clone formation assay was carried out as follows: the transfected cells with a density of 3,000 cells per well were seeded into six-well plates cultured for 2 weeks. The plates were fixed in 4% paraformaldehyde for 20 min and stained with 1% crystal violet for 20 min. The clone formation rate was calculated under a microscope, and more than 50 cell clones were considered one colony formation.

### 2.8 Transwell assay *in vitro*

For the migration assay, treated cells were seeded onto the upper chamber at 1 × 10^5^ cells per well, and 600 μl complete medium supplemented with 10% FBS was added to the bottom chamber. After 24 h of incubation, the non-migrating cells on the upper surface were removed and cells that migrated into the lower surface were fixed, stained, and then counted in five randomly selected microscopy fields. *In vitro* invasion assay was additionally pre-coated with Matrigel, and the rest of the steps was the same.

### 2.9 Prediction of associated miRNA of PTPN12

The candidate-associated miRNAs of PTPN12 were predicted using TargetScan (http://www.Targetscan.org/) and Omicsbean (http://www.omicsbean.cn/). Briefly, the human species was selected and PTPN12 was input into as the target gene. This identified five miRNAs, including miR-338-3p, miR-506-3p, miR-194-5p, miR-146b-3p, and miR-124-3p. Primers of the five miRNAs were designed, respectively, and checked for their expression in LSCC cells by qRT-PCR. Primer sequences are listed in Table A1. cDNA was reverse transcribed from total RNA samples using Transcriptor First Strand cDNA Synthesis Kit (Roche, Vaud, Switzerland) according to the manufacturer’s protocol. The qRT-PCR was performed using GoTaq®qPCR Master Mix (Promega, USA) according to the manufacturer’s manual by GoTaq 2-Step RT-qPCR System. qRT-PCR was performed as follows: 95°C for 2 min; 40 cycles at 95°C for 15 s, 59°C for 30 s, and 72°C for 30 s. (The annealing temperature varied for each set of primers.) The relative expression of miRNAs was quantified using 2^−△△Ct^ with U6 as an endogenous control. The experiments were assayed in triplicate. The expression levels for both miRNAs and PTPN12 on 60 paired samples were detected, and Spearman’s rank correlation was fitted to model the correlation analysis between miRNAs and PTPN12, and *P* < 0.05 was considered statistically significant. Only negative correlations were considered for further analyses. The miR-146b-3p showed an inverse correlation with PTPN12 expression, and other miRNAs are uncorrelated. Finally, miR-146b-3p was selected for subsequent experiments for its inhibition role in luciferase reporter assay.

### 2.10 Transfection of miRNA mimic and miRNA inhibitor

LSCC cells were cultured by antibiotics-free medium 1 week prior to transfection. NC, miR-146b-3p mimic, and miR-146b-3p inhibitor were purchased from genepharma (Shanghai, China) and diluted as the instruction indicated. The remaining steps were identical to the transfection assay. Transfection efficiency was measured by qRT-PCR analyses 24 h post-transfection. Cells were sequentially termed miR-146b-3p mimic, miR-146b-3p inhibitor, and miR-NC (served as a negative control).

### 2.11 The expression of PTPN12 after transfected with miR-146b-3p inhibitor and mimic used qRT-PCR and WB assay

qRT-PCR amplification steps were the same as previously described. Harvested cells were washed with pre-cooled PBS and lysed with RIPA buffer on ice for 30 min, and then the mixture was broken with an ultrasound shaker for full lysis followed by heating for 5 min at 99°C. Protein samples from equal amount were separated by pre-formed separating glue and concentrated glue (80 V, 30 min. 120 V, 1 h), transferred onto polyvinylidene difluoride membranes, and blocked with 5% non-fat milk. Membranes with anti-PTPN12 (Abcam, Cambridge, UK, dilution 1:1,000) antibody or ACTB monoclonal antibody were incubated overnight at 4°C on shaker and then incubated with horseradish peroxidase-labeled goat anti-rabbit secondary antibody at room temperature for 1 h. At last, stripes were detected by ECL chemiluminescence.

### 2.12 Luciferase plasmid construction and Dual-luciferase reporter assay

Specific binding sites of the miR-146b-3p identify PTPN12 3′-UTR were acquired from the online software (http://www.targetscan.org/), and subjected to PCR amplification. Next, the pmirGLO PTPN12 3′-UTR wild-type vector and mutant-type vector (mutagenic primer were designed using NEBaseChanger™ V1.2.8, http://nebasechanger.neb.com/) were constructed by Sangon Biotech followed by plasmid extraction according to the protocol. Luciferase reporter gene plasmids and NC, miR-146b-3p mimic, and miR-146b-3p inhibitor were transfected into AMC-HN-8 and 293T cells with Lipofectamine 2000. Two days posttransfection, the firefly and renilla luciferase activities were detected separately by a Dual-Luciferase Reporter Assay System (Promega, USA) on a microplate reader (TECAN, Switzerland). The ratio of firefly luciferase/Renilla luciferase was calculated as the promoter activity.

### 2.13 The gain and loss functional experiments of miR-146b-3p

For the proliferation, migration, and invasion assay, the experiment steps were identical to the procedures described previously.

### 2.14 The functional experiments in LSCC cells co-transfected by pcDNA3.1-PTPN12 and miR-146b-3p mimic

The MTS and transwell assay were performed to evaluate the synergistic effect of PTPN12 and miR-146b-3p, the functional experiment steps were identical to the procedures described previously.

### 2.15 Prediction of downstream target genes of PTPN12

We utilized online bioinformatics prediction software STRING database (https://cn.string-db.org/) and GeneCards database (https://www.genecards.org/) to determine potential downstream target genes of PTPN12. EGFR and ERBB2 that were common between the two databases were selected as the downstream-regulation target genes.

### 2.16 The qRT-PCR and WB analyses were performed to evaluate the mRNA and protein expression levels of EGFR and ERBB2

The qRT-PCR and WB analyses were used to assess the mRNA and protein expression levels of EGFR and ERBB2 in AMC-HN-8 cell with PTPN12 and miR-146b-3p over-expression. Primer sequences are listed in Table A1. The experiment steps were identical to the procedures described previously.

### 2.17 Statistical analysis

SPSS21.0 (IBM-SPSS Inc., Chicago, IL, USA) was used for statistical analysis. Data were expressed as mean ± standard, and Student’s *t*-test or Wilcoxon rank-sum was performed to analyze the differences between groups. The relationship between expression level and clinical parameters was verified by an independent-sample *t* test. All tests were two-sided with *P* < 0.05 considered statistical significance.

## 3 Results

### 3.1 PTPN12 expression is down-regulated in LSCC tissues and cell lines

**Figure 1:**
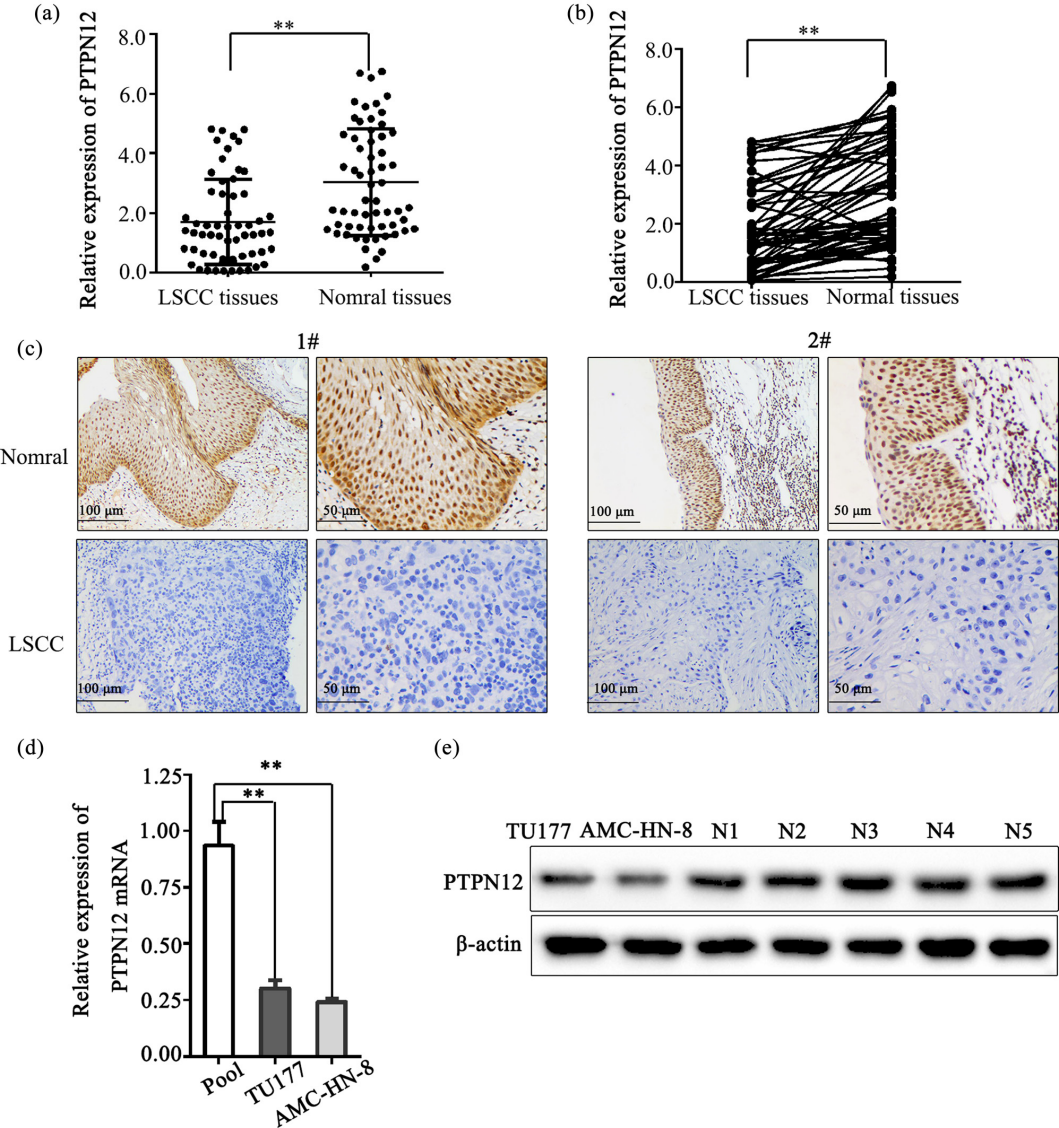
PTPN12 expression in LSCC tissues and cell lines. (a). Relative expression of PTPN12 mRNA in LSCC tiusses and paired paracancerous tissues. (b). Comparison of the relative expression of PTPN12 mRNA between tumor tissues and paired adjacent normal tissues. (c). PTPN12 protein expression in LSCC tissues and paired paracancerous tissues from two patients. (d). Relative expression of PTPN12 mRNA in two laryngeal cancer cell lines. The Pool represents mean expression level of PTPN12 mRNA in 20 para-cancer normal tissues. (e) The protein expression levels of PTPN12 in LSCC cells and five normal tissue samples. Data are presented as the mean ± SD. Student*s *t*-test was used for *P* value assessment. *P < 0.05, **P < 0.01.

The PTPN12 mRNA expression level was significantly lower in LSCC tissues compared to paired paracancerous tissues (*P* < 0.01) (Figure 1a and b). Correlation analysis between PTPN12 expression and clinicopathological features indicated that PTPN12 mRNA expression level was lower in patients with poor differentiation than that with well/moderate differentiation (*P* < 0.05) (Table 1). Immunohistochemistry showed that the PTPN12-positive protein expression frequencies were 33.3% (20/60) and 81.7% (49/60) in LSCC tissues and paired adjacent normal tissues, respectively, which were significant (*P* < 0.01) (Figure 1c) (Table 2). In LSCC patients, PTPN12-positive protein expression was significantly associated with the TNM stage (*P* = 0.018) (Table 3). The same decreasing tendency of mRNA expression was observed in AMC-HN-8 and TU177 cells using qRT-PCR (Figure 1d). “Pool” was defined as an average expression of 20 cases corresponding to adjacent normal tissues. WB (Figure 1e) analysis confirmed decreased PTPN12 protein expression in LSCC cell lines and elevated PTPN12 protein expression in normal tissues. AMC-HN-8 was chosen for the subsequent research object due to its lower expression of PTPN12.

**Table 2:** PTPN12-positive protein expression frequency in LSCC tissues

Tissues	*N*	PTPN12-positive protein expression		
		*n* (%)	*χ* ^2^	*P*
Tumor	60	20 (33.3)	28.679	0.000
Normal	60	49 (81.7)		

**Table 3:** Expression of PTPN12 protein in LSCC and its relationship with clinicopathological features

Characteristics	*n* (%)	Expression level of PTPN12 protein	
		*χ* ^2^	*P*
Age (years)			
<60	10(47.6)	2.976	0.765
≥60	10(25.6)		
Smoking			
No	4(66.7)	1.875	0.171
Yes	16(29.6)		
TNM stage			
Ⅰ + Ⅱ	14(48.3)	5.640	0.018
Ⅲ + Ⅳ	6(19.4)		
Lymph node metastasis			
N0	9(25.0)	2.813	0.094
N1/2/3	11(45.8)		
Pathological differentiation			
Well/moderate	16(31.4)	0.147	0.701
Poor	4(44.4)		

### 3.2 Overexpression of PTPN12 inhibits LSCC cell proliferation, migration, and invasion abilities

**Figure 2:**
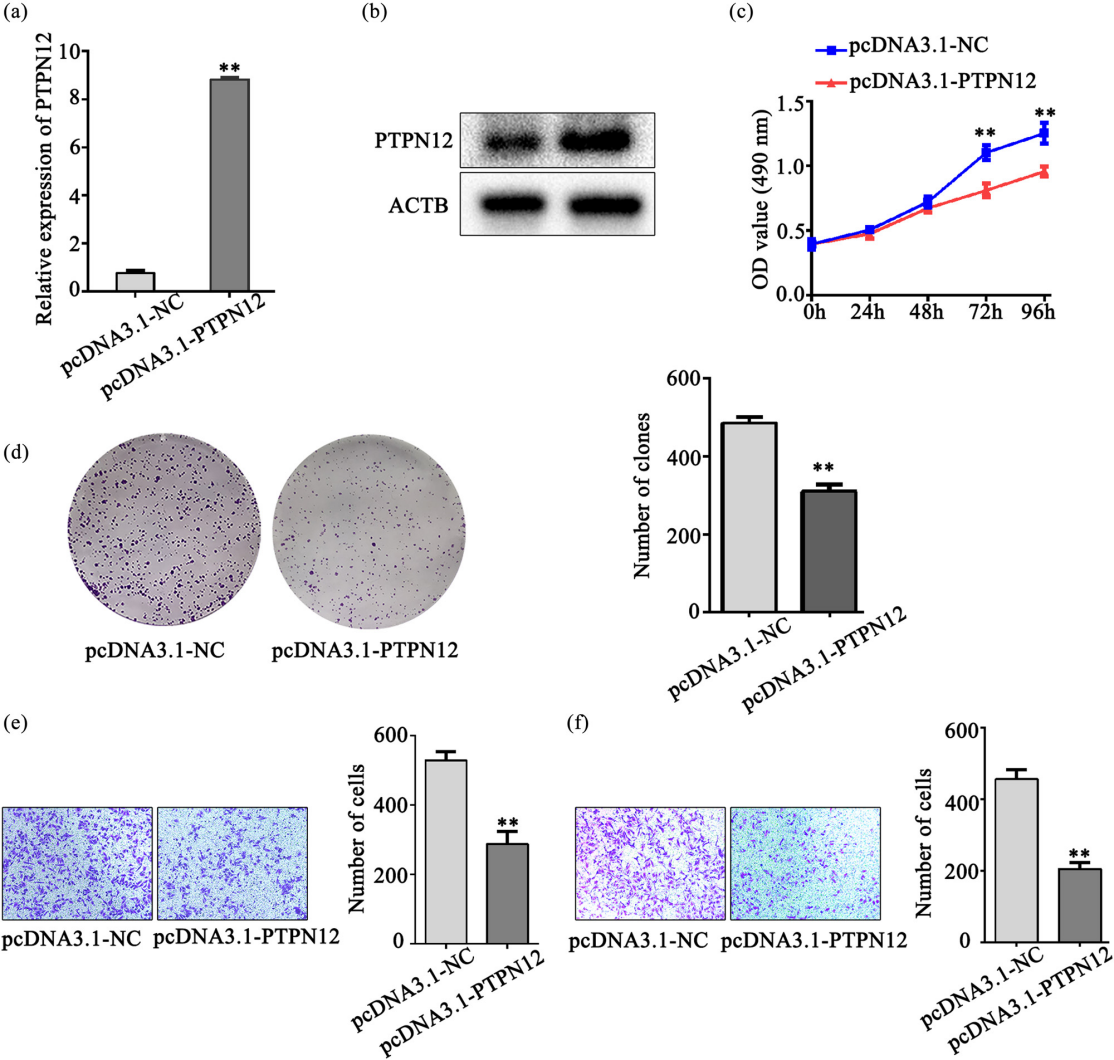
The biological function of AMC-HN-8 overexpressing PTPN12 *in vitro*. (a). PTPN12 overexpression efficiency was verified by qRT-PCR analysis in AMC-HN-8 cells after transfection with pcDNA3.1-PTPN12 or pcDNA3.1 empty vector. (b). PTPN12 overexpression efficiency was verified by WB analysis in AMC-HN-8 cells after transfection with pcDNA3.1-PTPN12 or pcDNA3.1 empty vector. (c). The MTS assay was used to determine the cell proliferation ability. PTPN12 overexpression led to marked inhibition of tumor cell proliferation capacity than cell transfected with the pcDNA3.1 empty vector at the indicated time points. (d). A colony formation assay was carried out to analyse the cell proliferation ability. Cells transfected with pcDNA3.1-PTPN12 had decreased colony formation. (e–f). Transwell migration and invasion assays demonstrated that overexpressing PTPN12 notably decreased cell migration ability and invasion ability compared with control cells. Data are presented as the mean ± SD of three independent experiments. **P < 0.01.

Considering the decreased expression of PTPN12 in LSCC tissues and cell lines, AMC-HN-8 was transfected with pcDNA3.1-PTPN12 and pcDNA3.1-NC. The overexpression efficiency was verified by qRT-PCR and WB. As shown in Figure 2a and b, the relative expression of PTPN12 was significantly increased following PTPN12 overexpression. MTS and clone formation results showed that PTPN12 overexpression led to marked inhibition of tumor cell proliferation capacity *in vitro* (Figure 2c and d). In the transwell migration assay, LSCC cell overexpressing PTPN12 showed a reduced migratory capacity compared to the control group (Figure 2e). As shown in Figure 2f, LSCC cell invaded through artificial membranes in the pcDNA3.1-PTPN12 group was apparently lower.

### 3.3 miR-146b-3p is up-regulated and negatively correlates with PTPN12 in LSCC tissues

**Figure 3:**
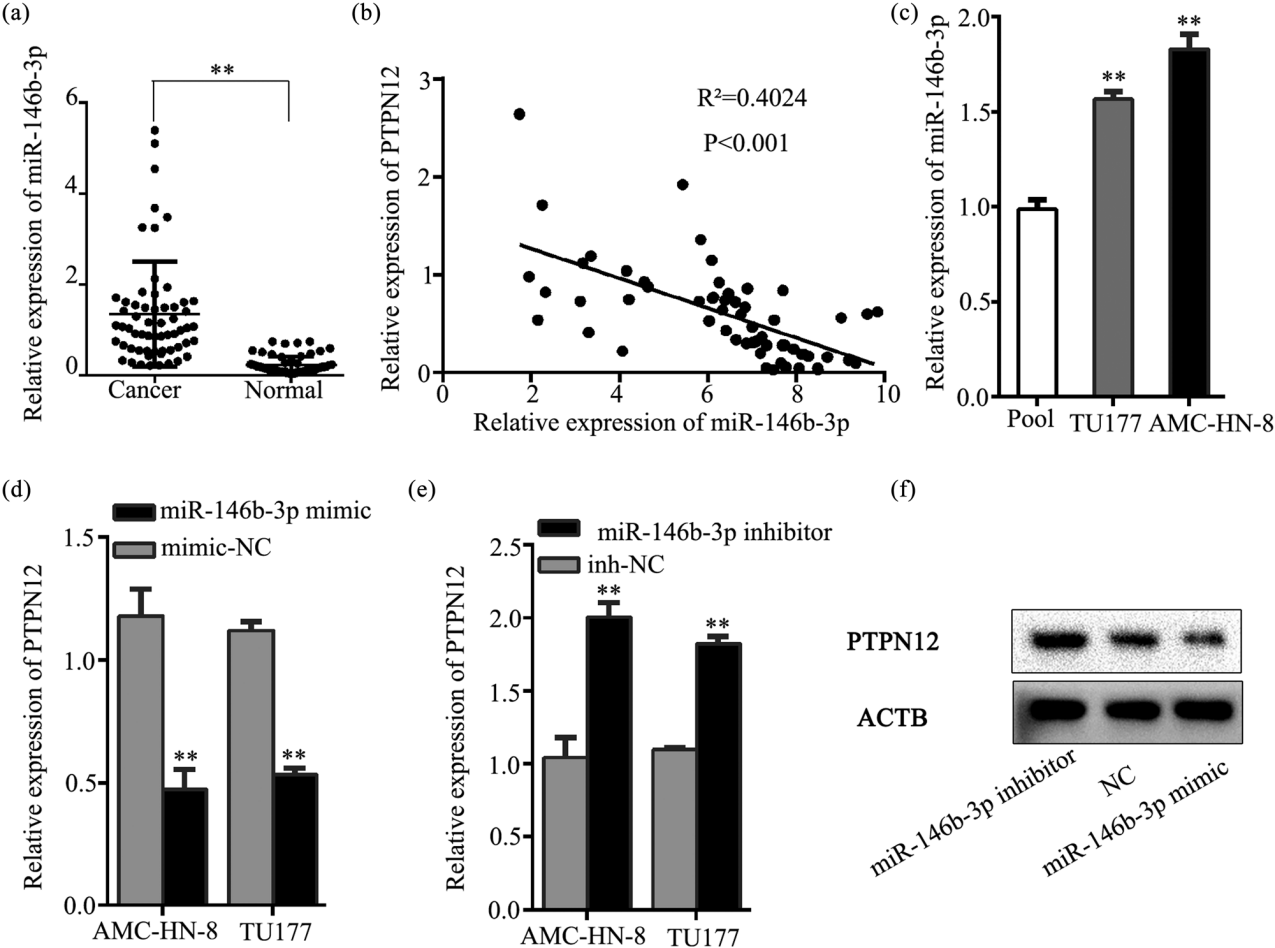
The correlation of PTPN12 expression and miR-146b-3p expression. (a). Relative expression of miR-146b-3p in LSCC tiusses and paired paracancerous tissues. (b). *Pearsons* correlation analysis was performed to explore the correlation between the expression of PTPN12 and miR-146b-3p. (c). Relative expression of miR-146b-3p in two laryngeal cancer cell lines. The Pool represents mean expression level of miR-146b-3p in 20 para-cancer normal tissues. (d–e). qRT-PCR was carried out to analyse the relative expression of PTPN12 in LSCC cells transfected with miR-146b-3p mimic and inhibitor. (f). WB was carried out to analyse the PTPN12 protein expression in AMC-HN-8 cell transfected with miR-146b-3p mimic and inhibitor. **P < 0.01.

**Figure A1:**
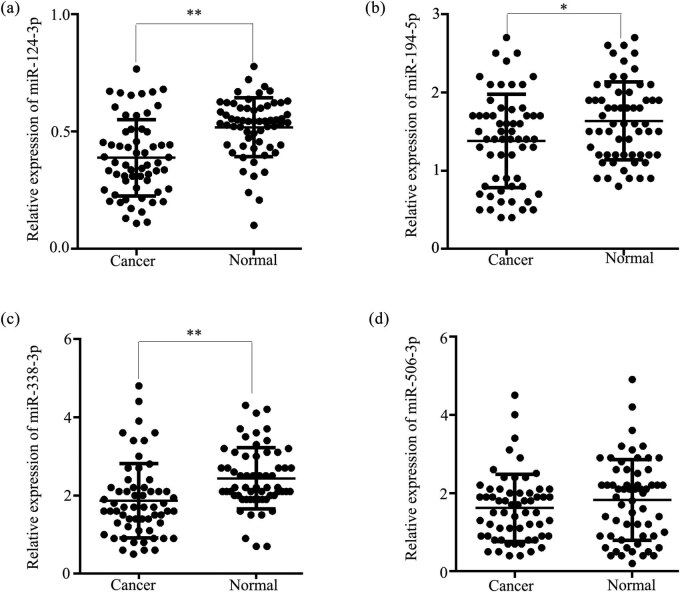
Relative expression of miR-124-3p in LSCC tiusses and paired paracancerous tissues. (b). Relative expression of miR-194-5p in LSCC tiusses and paired paracancerous tissues. (c). Relative expression of miR-338-3p in LSCC tiusses and paired paracancerous tissues. (d). Relative expression of miR-506-3p in LSCC tiusses and paired paracancerous tissues. *P < 0.05, **P < 0.01.

To determine the effect of miRNAs on PTPN12 expression, qRT-PCR was performed in LSCC tissues and paired normal tissues. The result revealed that the expression of miR-146b-3p in LSCC tissues was significantly higher for LSCC tumor tissues versus normal tissues (Figure 3a), whereas miR-124-3P, miR-194-5P, and miR-338-3P were highly expressed in normal tissues, and no differential expression was observed for miR-506-3P (Figure A1). Pearson correlation analysis indicated a significant inversely correlation between miR-146b-3p and PTPN12 expression (*R* ^2^ = 0.4024, *P* < 0.01) (Figure 3b). As shown in Figure 3c, the expression of miR-146b-3p in AMC-HN-8 and TU177 cells was significantly higher using qRT-PCR. qRT-PCR analyses of AMC-HN-8 and TU177 cells indicated a significant down-regulation of PTPN12 upon miR-146b-3p overexpression (Figure 3d). Likewise, a marked up-regulation of PTPN12 expression following miR-146b-3p knockdown was observed (Figure 3e). Furthermore, a similar result was observed by WB experiment in AMC-HN-8, which was consistent with the qRT-PCR data (Figure 3f). All the results above demonstrated that up-regulation of miR-146b-3p may be linked to LSCC progression.

### 3.4 PTPN12 is a direct target of miR-146b-3p in LSCC cells

**Figure 4:**
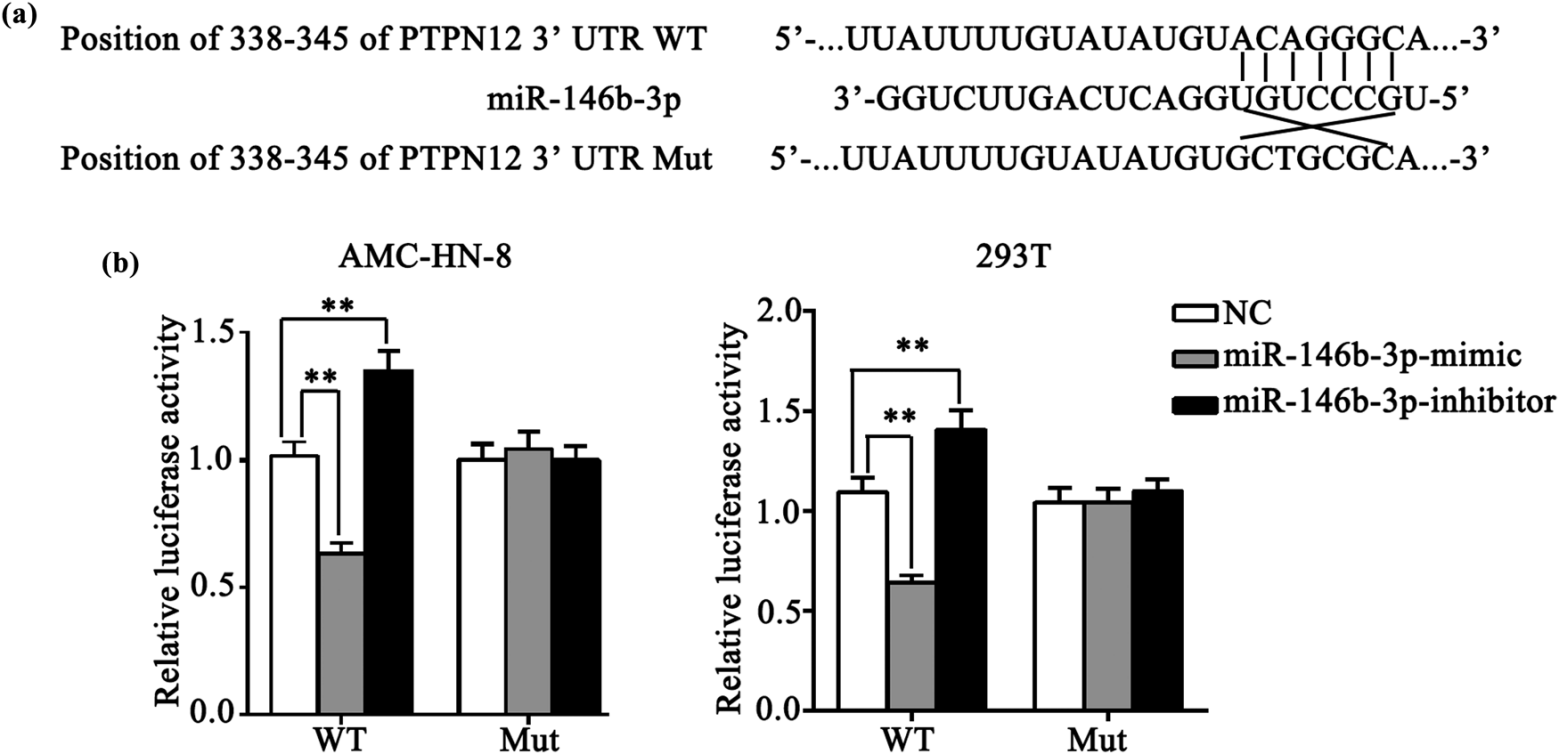
MiR-146b-3p directly targets PTPN12 in LSCC cell. (a). pmirGLO-PTPN12-3′-UTR-WT and pmirGLO-PTPN12-3′-UTR-Mut luciferase reporter plasmids with the miR-146b-3p binding site. (b). Luciferase activity was significantly decreased in AMC-HN-8 and 293 T cells co-transfected with PTPN12-3′-UTR-WT and miR-146b-3p mimic in vitro. **P < 0.01.

To verify the role of the PTPN12 3′-UTR in miR-146b-3p targeting, we constructed pmirGLO-PTPN12-3′-UTR-WT and pmirGLO-PTPN12-3′-UTR-Mut luciferase reporter plasmids with the miR-146b-3p-binding site (Figure 4a). AMC-HN-8 cell and 293T cell were co-transfected with control empty vector, PTPN12-3′-UTR-WT or PTPN12-3′-UTR-Mut and miR-146b-3p mimic or miR-146b-3p inhibitor. As can be seen in Figure 4b, compared with the NC group, the luciferase activity was significantly decreased in AMC-HN-8 and 293T cells co-transfected with PTPN12-3′-UTR-WT and miR-146b-3p mimic, whereas the activity was significantly increased in cells co-transfected with PTPN12-3′-UTR-WT and miR-146b-3p inhibitor.

### 3.5 The impact of miR-146b-3p on the proliferation, migration, and invasion of LSCC cells

**Figure 5:**
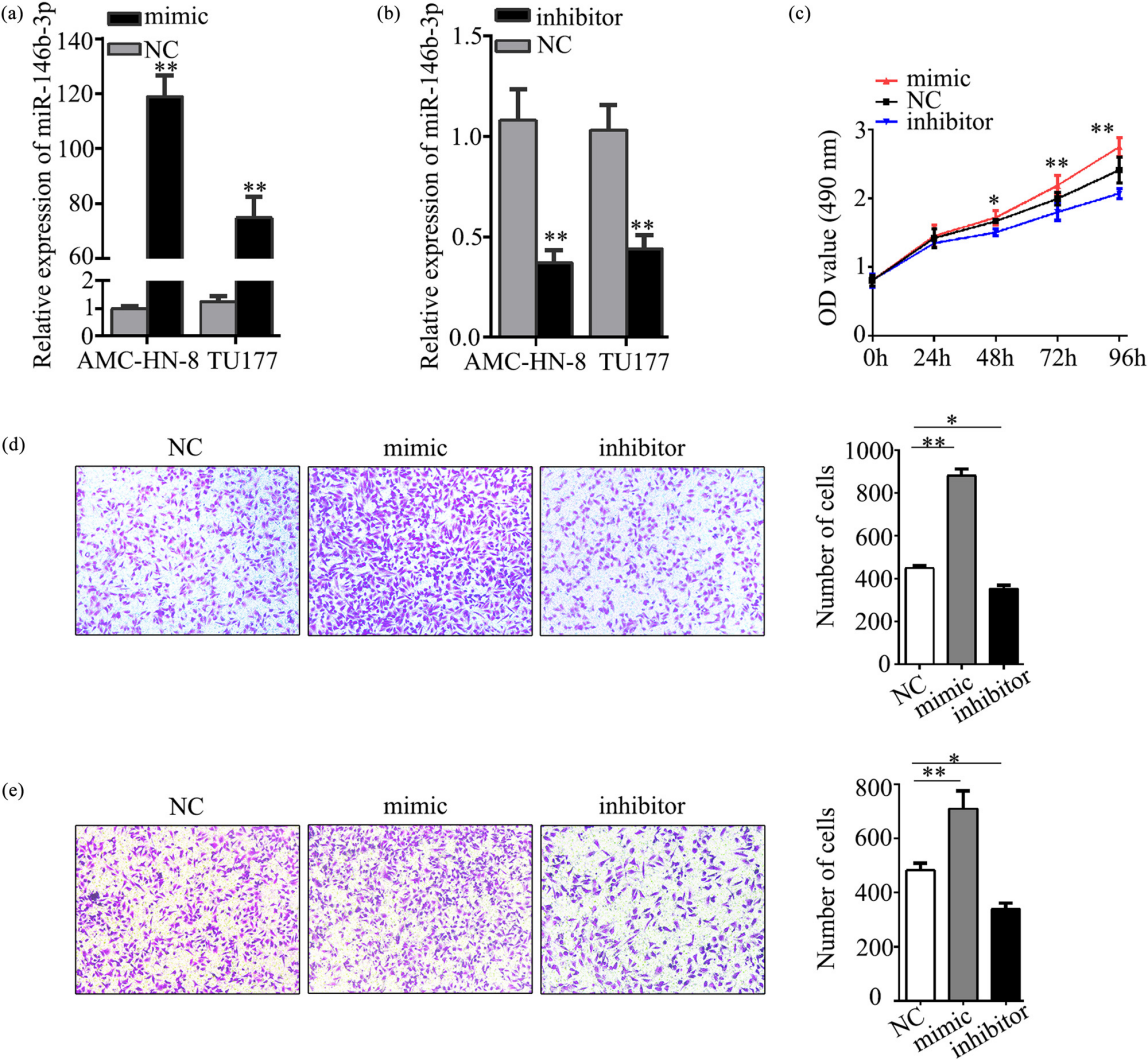
The biological function of miR-146b-3p *in vitro*. (a and b). Transfection efficiency was verified by qRT-PCR analysis in AMC-HN-8 and TU177 cells transfected with miR-146b-3p mimic and inhibitor. (c). The MTS assay was used to determine the cell proliferation ability. The miR-146b-3p inhibitor led to marked inhibition of tumor cells proliferation capacity than cells transfected with the miR-146b-3p mimic at the indicated time points. (d and e). Down-regulated miR-146b-3p expression dramatically decreased the ability of LSCC cell migration ability and invasion ability in vitro. Data are presented as the mean ± SD of three independent experiments. *P < 0.05, **P < 0.01.

To ascertain the functional consequences of altered miR-146b-3p expression, we examined the effect of loss or gain of function in AMC-HN-8 cells due to its higher expression of miR-146b-3p. Transfected with miR-146b-3p mimic or inhibitor and verified transfection efficiency were performed. The qRT-PCR results indicated that in comparison with mimics NC and inhibitor NC, the miR-146b-3p expression was significantly up-regulated in mimic and down-regulated in the inhibitor group (Figure 5a and b). Functional miR-146b-3p experiments were designed into three groups: NC, miR-146b-3p mimic, and miR-146b-3p inhibitor. The MTS assay indicated that in the miR-146b-3p inhibitor group, LSCC cell proliferation capability started to decrease from 48 h, and significant inhibition was observed in 72 and 96 h (Figure 5c). In transwell migration and invasion assays, miR-146b-3p inhibitor cells displayed diminished migration and invasion abilities compared to NC and miR-146b-3p mimic groups (Figure 5d and e). Conversely, overexpressing miR-146b-3p had an opposite result (Figure 5d and e).

### 3.6 Synergism between miR-146b-3p and PTPN12

**Figure 6:**
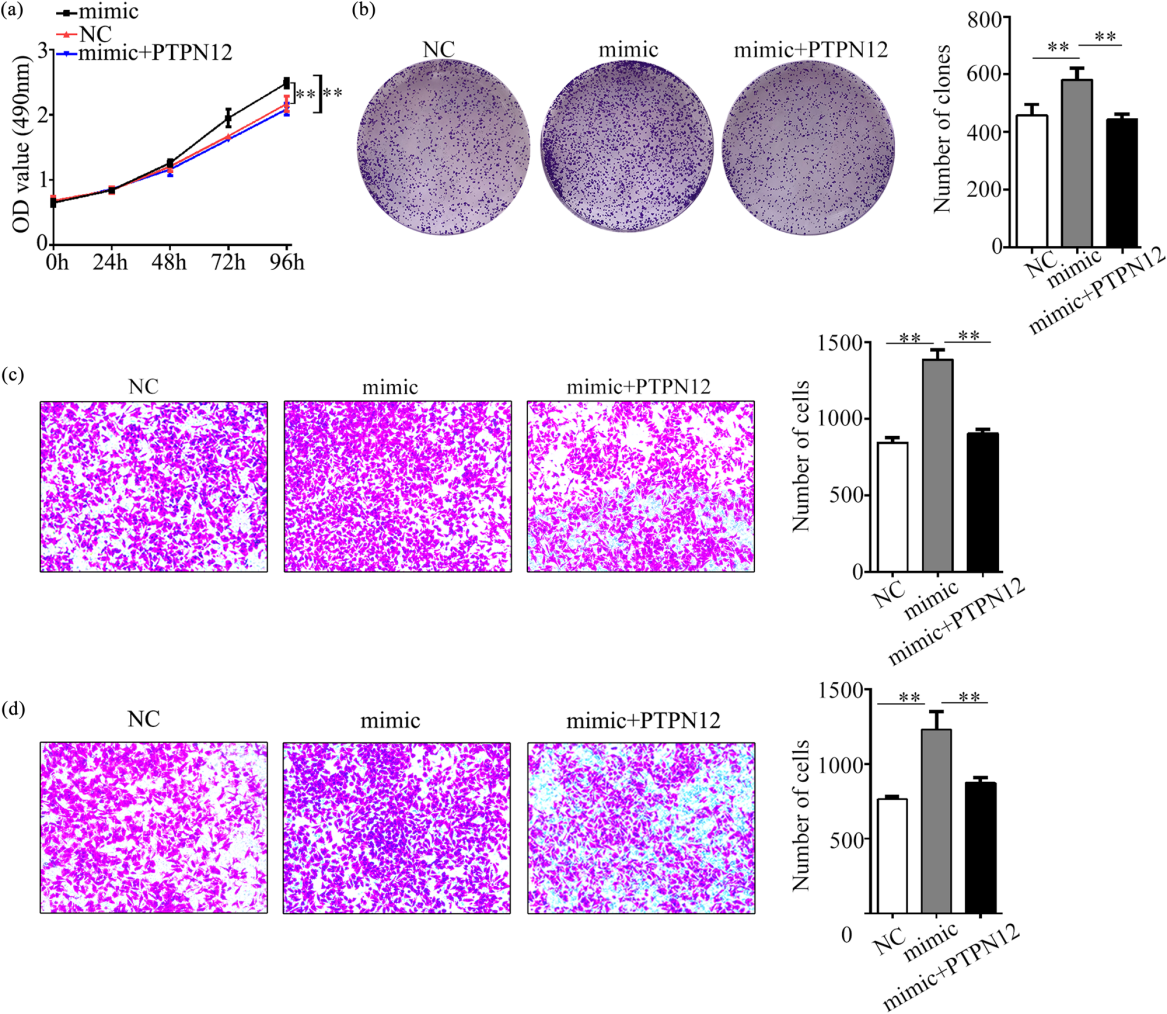
Synergistic effect of miR-146b-3p and PTPN12 on biological behavior of laryngeal cancer cell. (a). Growth curve of AMC-HN-8 after cotransfected with miR-146b-3p mimic and pcDNA3.1-PTPN12 was determined by MTS assay. (b). A colony formation assay of AMC-HN-8 cell after cotransfected with miR-146b-3p mimic and pcDNA3.1-PTPN12. (c). Migration assay and (d). Invasion assay of AMC-HN-8 cell after cotransfected with miR-146b-3p mimic and pcDNA3.1-PTPN12. Data are presented as the mean ± SD of three independent experiments. *P < 0.05, **P < 0.01.

To further investigate the synergism, we also performed co-transfecting experiments, which were divided into three groups named pcDNA3.1-NC, pcDNA3.1-PTPN12, and pcDNA3.1-PTPN12 + miR-146b-3p mimic. Our results revealed that overexpression of PTPN12 markedly inhibited the proliferation, migration, and invasion abilities of AMC-HN-8 (Figure 6a–d), whereas the inhibitory effects were partially recovered by co-transfection with pcDNA3.1-PTPN12 + miR-146b-3p mimic.

### 3.7 Prediction of potential downstream target genes of PTPN12

**Figure 7:**
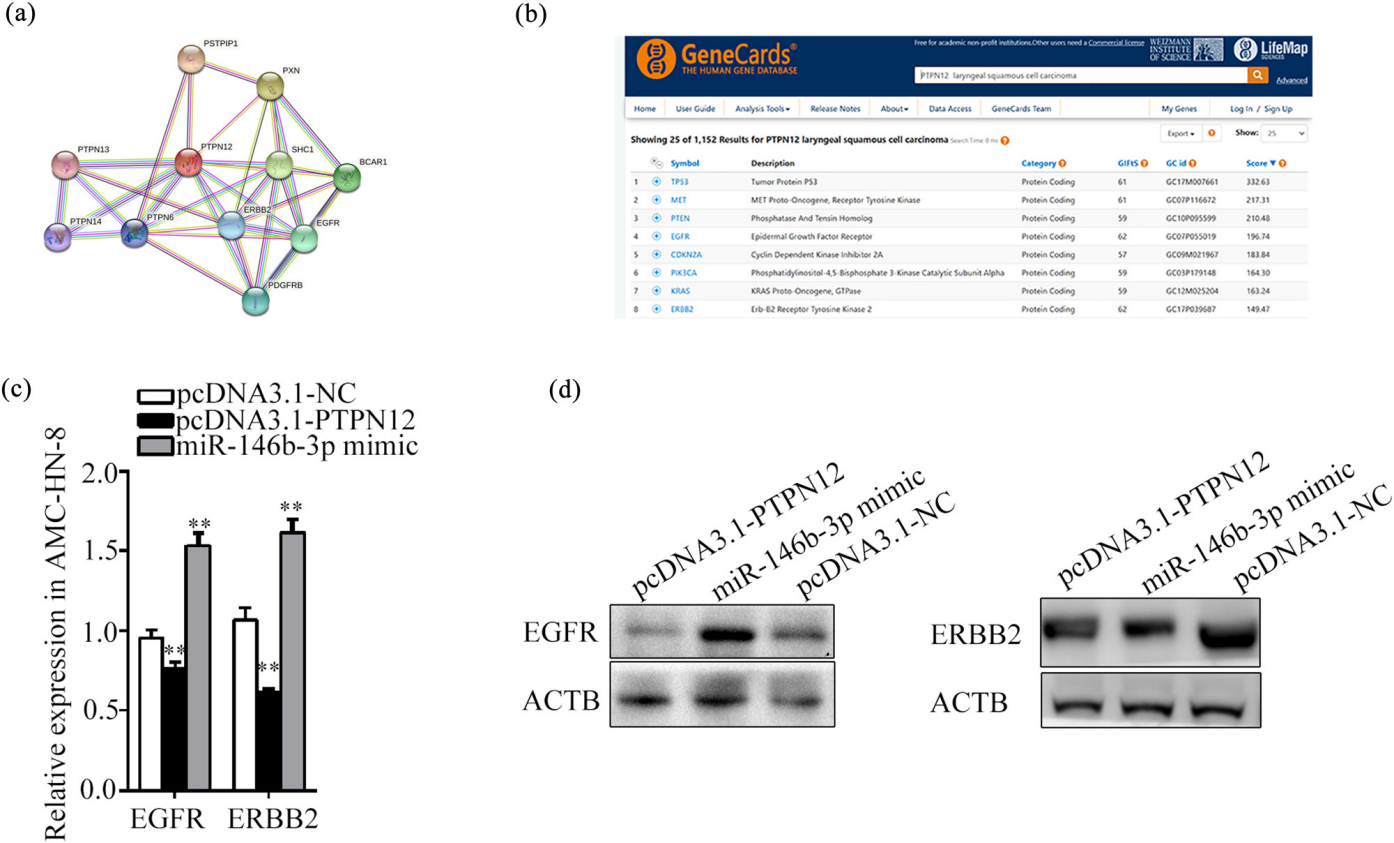
Prediction of downstream target genes of PTPN12. (a). Prediction potential downstream targets of PTPN12 was performed by the online software STRING. (b). Prediction potential downstream targets of PTPN12 was performed by the online software GeneCards database. (c). Relative expression of EGFR and ERBB2 in AMC-HN-8 after transfected with miR-146b-3p mimic and pcDNA3.1-PTPN12 was determined by qRT-PCR assay. (d). Relative expression of EGFR and ERBB2 in AMC-HN-8 after transfected with miR-146b-3p mimic and pcDNA3.1-PTPN12 was determined by WB assay. Data are presented as the mean ± SD of three independent experiments. **P < 0.01.

The prediction results showed that EGFR and ERBB2 were the potential downstream targets of PTPN12 (Figure 7a and b). Overexpression of PTPN12 down-regulated the mRNA expression levels of EGFR and ERBB2 (*P* = 0.000) in AMC-HN-8, as detected by the qRT-PCR method (Figure 7c). Meanwhile, their expression levels showed obvious elevation after overexpression of miR-146b-3p. The protein expression levels of EGFR were consistent with the mRNA levels, as evidenced by the WB analysis; however, the protein and mRNA expression of ERBB2 was inconsistent (Figure 7d).

## 4 Discussion

PTPs, also known as PTP-PEST (proline, glutamic acid, serine, and threonine), act as an inhibitor of protein tyrosine kinase signaling pathway and involves multiple tumor inhibition [20]. PTPN12, mapped to chromosome 7q11.23, is a member of the PTP family and is expressed in multiple cell types. This study provides the first description of the expression and clinical significance of PTPN12 in LSCC and provides evidence suggesting that PTPN12 functions as a tumor suppressor in the development and progression of LSCC. Then, we further investigated the upstream regulatory miRNA of PTPN12. We conclude that miR-146b-3p targets PTPN12 and functions as an oncogene in LSCC. Recent investigations have indicated that PTPN12 exerts its role as a tumor suppressor by regulating various signaling pathways in multiple cancers. For instance, in breast cancer, PTPN12 inhibits the malignant transformation of human mammary normal epithelial by down-regulation of growth factor receptor signaling pathway. Approximately 23% of breast cancer patients exhibit low or no expression of PTPN12, particularly in triple-negative breast cancer patients, which predicts a poor prognosis [21]. A study shows that the expression of PTPN12 is much lower in non-squamous cell lung cancer tissues than in normal lung tissues; high expression of PTPN12 predicts longer disease-free survival and overall survival [22]. To investigate the potential effect of PTPN12 in LSCC, first, we determined the differential expression levels of PTPN12 in LSCC tissues and corresponding adjacent normal tissues. We found that PTPN12 is down-regulated in LSCC tissues and cell lines, and its low expression was correlated with the advanced TNM stage in LSCC tissues, suggesting that the down-regulation of PTPN12 may contribute to the progression of LSCC.

Piao et al. show that expression of PTPN12 is negatively correlated with tumor size, pathologic grading, TNM stage, and tumor recurrence in bladder transitional cell carcinoma. In addition, inhibition expression of PTPN12 remarkably enhances the proliferative, migratory, and invasive capacities of bladder carcinoma cells both *in vitro* and *in vivo* [23]. Consistent with the previous results, over-expression of PTPN12 led to an inhibition of proliferative, migratory, and invasive capacities in the LSCC cell line. These results indicated that PTPN12 may play essential roles in modulating LSCC carcinogenesis, migration, and invasion. Based on these results, it can be concluded that PTPN12 may function as a tumor suppressor gene.

miRNAs are short non-coding RNAs that are linked in a variety of post-transcriptional regulations, leading to the translational inhibition or destabilization of the target mRNA by binding to its 3′-UTR region. As a growing number of cancer-related miRNAs are identified, numerous studies have been performed due to their dual roles as oncomiRNAs or anti-tumor genes. The regulatory mechanism of miRNAs on tumor can be delineated as follows. Dysregulation of key enzymes Drosha and Dicer in miRNAs maturation is related to tumorigenesis. Studies indicate that oncogene c-Myc regulates the expression of Drosha by binding to drosha, leading to the inhibition of miRNAs, which promotes tumorigenesis [24]. Dysregulated miRNA acted as a tumor suppressor gene. This mechanism is verified by studies about miR-34 family and cancers. miR-34a plays an inhibitory role by targeting Bcl-2 and SIRT1 and down-regulating their expression in breast cancer [25]. Due to their target genes were mainly focused on EMT, miR-200 family are associated with tumor metastasis, for instance, zinc-finger E-box-binding homeobox 1 (ZEB1) and transforming growth factor β1 (TGFβ1) [26,27]. Dysregulation of miRNAs with oncogenic function. Dysregulation of miR-21, miR-155, and miR-221 contributes to tumor development and progression [28,29]. In search of miRNAs that regulate PTPN12, we used online software and co-analyzed the expression changes in candidate miRNAs and PTPN12 expressions. The miR-146b-3p was selected for its inverse correlation with PTPN12 expression and its inhibition role in a luciferase reporter assay.

The role of miR146b-3p is controversial, and it may play various roles in different tumors. In cervical cancer, miR-146b-3p serves as oncogene by promoting cervical cancer cell proliferation, migration, and invasion capacities [30]. Elevation in miR-146b-3p acts as an independent predictor of central lymph node metastases in papillary thyroid carcinoma [31]. Our studies are the first to demonstrate the tumor-promoting effects of miR146b-3p in LSCC. Elevated miR146b-3p expression was found in LSCC tissues and *PTPN12* was identified as a target mRNA. Furthermore, inhibition of miR-146b-3p suppressed LSCC cell proliferation, migration, and invasion. The tumor-promoting effect of miR146b-3p could be restored by the overexpression of PTPN12 in LSCC cells. Our results show an essential role of miR-146b-3p in LSCC pathogenesis and provide an emerging effective target in LSCC therapy.

PTPN12 and downstream proteins interaction analysis was predicted based on STRING and GeneCards, EGFR and ERBB2 were selected for targets of PTPN12. The ErbB/HER protein kinase family contains four structurally associated receptor tyrosine kinases including ErbB-1/HER1/EGFR, ErbB-2/HER2, ErbB-3/HER3, and ErbB-4/HER4 [32]. EGFR is overexpressed in bladder, breast, head and neck, non-small-cell lung, and prostate cancers, indicating that EGFR is involved in the malignant biological behavior of tumor cells [33,34,35,36]. ERBB2 is expressed at a low level and almost undetectable in normal tissues [37], but it is found to be overexpressed in multiple tumors, such as breast cancer, ovary carcinoma, prostate carcinoma, and non-small-cell lung cancer [38,39,40]. ERBB2 initiates downstream MAPK/ERK kinase signaling by forming heterodimers with ERBB1. The MAPK/ERK signaling pathway has been extensively studied due to its involvement in tumorigenesis, proliferation, evasion, angiogenesis, apoptosis, adhesion, and malignant transformation. In the present study, we found that up-regulation of PTPN12 significantly suppressed EGFR expression, and miR-146b-3p mimic significantly up regulated the EGFR expression. However, ERBB2 was in fact the opposite; up-regulation of PTPN12 and miR-146b-3p mimic suppressed its expression at protein levels, which was shown inconsistencies with its mRNA expression levels. The inconsistency between mRNA and protein expression may be due to posttranscriptional regulation or a delay in protein expression. This provided further evidence that miR-146b-3p mimic can act as an oncogene in LSCC through the regulation of EGFR expression. Conversely, PTPN12 acts as a tumor suppressor gene through the regulation of EGFR expression. However, further experiments are required to rigorously test these hypotheses.

## 5 Conclusions

In conclusion, the down-regulation of PTPN12 is associated with the up-regulation of miR-146b-3p in LSCC. Moreover, PTPN12 serves as a tumor suppressor gene by regulating the proliferation, migration, and invasion of LSCC cells. miR-146b-3p/PTPN12 axis is expected to be a novel therapeutic target in LSCC.

## Appendix

**Table A1:** Primer sequences of related genes for qRT-PCR

Gene	Application	Primer sequence
PTPN12	qRT-PCR	F: 5′-ACACCAAGTCCTACAACAC-3′
		R: 5′-GTCACAGCACCATCAGAG-3′
GAPDH	qRT-PCR	F: 5′-GAAGCTCGTCATCAATGGAAA-3′
		R: 5′-CCACTTGATGTTGGCAGGAT-3′
U6	RT	5′-CGAGCACAGAATCGCTTCACGAATTTGCGTGTCAT-3′
	qRT-PCR	F: 5′-GACACGCAAATTCGTG-3′
		R: 5′-GTGCAGGGTCCGAGGT-3′
miR-338-3p	RT	5′-GTCGTATCCAGTGCAGGGTCCGAGGTATTCGCACTGGATACGACATGCTGGA-3′
	qRT-PCR	F: 5′-GTGCAGGGTCCGAGGTATT-3′
		R: 5′-GCCGGTTGTTTTAGTGACTACGACCT-3′
miR-506-3p	RT	5′-GTCGTATCCAGTGCAGGGTCCGAGGTATTCGCACTGGATACGACCATTCCGT-3′
	qRT-PCR	F: 5′-GTGCAGGGTCCGAGGTATT-3′
		R: 5′-GCCGAGATGAGTCTTCCCACGGAATG-3′
miR-194-5p	RT	5′-GTCGTATCCAGTGCAGGGTCCGAGGTATTCGCACTGGATACGACACATTGTC-3′
	qRT-PCR	F: 5′-GTGCAGGGTCCGAGGTATT-3′
		R: 5′-GCCGAGGTGTACCTCAACGACAATGT-3′
miR-146b-3p	RT	5′-GTCGTATCCAGTGCAGGGTCCGAGGTATTCGCACTGGATACGACGCAGAA-3′
	qRT-PCR	F: 5′-GTGCAGGGTCCGAGGTATT-3′
		R: 5′-GCCGGGTCTTGACTCAGGTGTCCCGT-3′
miR-124-3p	RT	5′-GTCGTATCCAGTGCAGGGTCCGAGGTATTCGCACTGGATACGACATTCCGTG-3′
	qRT-PCR	F: 5′-GTGCAGGGTCCGAGGTATT-3′
		R: 5′-GCCGCCGTAAGTGGCGCACGGAAT-3′
EGFR	qRT-PCR	F: 5′-CAGCGCTACCTTGTCATTCAG-3′
		R: 5′-TCATACTATCCTCCGTGGTCA-3′
ERBB2	qRT-PCR	F: 5′-CGCTTTTGGCACAGTCTACA-3′
		R: 5′-TCCCGGACATGGTCTAAGAG-3′

## References

[1] Siegel RL, Miller KD, Wagle NS, Jemal A. Cancer statistics, 2023. CA Cancer J Clin. 2023;73(1):17–48. 10.3322/caac.21763. 36633525

[2] Sung H, Ferlay J, Siegel RL, Laversanne M, Soerjomataram I, Jemal A, et al. Global cancer statistics 2020: GLOBOCAN estimates of incidence and mortality worldwide for 36 cancers in 185 countries. CA Cancer J Clin. 2021;71(3):209–49. 10.3322/caac.21660. 33538338

[3] Rudolph E, Dyckhoff G, Becher H, Dietz A, Ramroth H. Effects of tumour stage, comorbidity and therapy on survival of laryngeal cancer patients: a systematic review and a meta-analysis. Eur Arch Otorhinolaryngol. 2011;268(2):165–79. 10.1007/s00405-010-1395-8. 20957488

[4] Li X, Wilmanns M, Thornton J, Köhn M. Elucidating human phosphatase-substrate networks. Sci Signal. 2013;6(275):rs10. 10.1126/scisignal.2003203. 23674824

[5] Hunter T. Signaling--2000 and beyond. Cell. 2000;100(1):113–27. 10.1016/s0092-8674(00)81688-8. 10647936

[6] Liang T, Li L, Cheng Y, Ren C, Zhang G. MicroRNA-194 promotes the growth, migration, and invasion of ovarian carcinoma cells by targeting protein tyrosine phosphatase nonreceptor type 12. Onco Targets Ther. 2016;9:4307–15. 10.2147/OTT.S90976. PMC4956060 27486333

[7] Li H, Yang F, Liu C, Xiao P, Xu Y, Liang Z, et al. Crystal Structure and Substrate Specificity of PTPN12. Cell Rep. 2016;15(6):1345–58. 10.1016/j.celrep.2016.04.016. 27134172

[8] Wei B, Zhu Y, Yang P, Han Y, Wang S, Wang X, et al. GABAB1e promotes the malignancy of human cancer cells by targeting the tyrosine phosphatase PTPN12. iScience. 2021;24(11):103311. 10.1016/j.isci.2021.103311. PMC8577127 34778730

[9] Kim O, Hwangbo C, Tran PT, Lee JH. Syntenin-1-mediated small extracellular vesicles promotes cell growth, migration, and angiogenesis by increasing onco-miRNAs secretion in lung cancer cells. Cell Death Dis. 2022;13(2):122. 10.1038/s41419-022-04594-2. PMC8826407 35136055

[10] Chen Z, Morales JE, Guerrero PA, Sun H, McCarty JH. PTPN12/PTP-PEST regulates phosphorylation-dependent ubiquitination and stability of focal adhesion substrates in invasive glioblastoma cells. Cancer Res. 2018;78(14):3809–22. 10.1158/0008-5472.CAN-18-0085. PMC6050134 29743287

[11] Di Leva G, Garofalo M, Croce CM. MicroRNAs in cancer. Annu Rev Pathol. 2014;9:287–314. 10.1146/annurev-pathol-012513-104715. PMC4009396 24079833

[12] He B, Zhao Z, Cai Q, Zhang Y, Zhang P, Shi S, et al. miRNA-based biomarkers, therapies, and resistance in Cancer. Int J Biol Sci. 2020;16(14):2628–47. 10.7150/ijbs.47203. PMC7415433 32792861

[13] Wang J, Yuan Y, Tang L, Zhai H, Zhang D, Duan L, et al. Long non-coding RNA-TMPO-AS1 as ceRNA binding to let-7c-5p upregulates STRIP2 expression and predicts poor prognosis in lung Adenocarcinoma. Front Oncol. 2022;12:921200. 10.3389/fonc.2022.921200. PMC9237420 35774125

[14] Sabit H, Cevik E, Tombuloglu H, Abdel-Ghany S, Tombuloglu G, Esteller M. Triple negative breast cancer in the era of miRNA. Crit Rev Oncol Hematol. 2021;157:103196. 10.1016/j.critrevonc.2020.103196. 33307198

[15] Cheng Y, Liu W. MicroRNA-503 serves an oncogenic role in retinoblastoma progression by directly targeting PTPN12. Exp Ther Med. 2019;18(3):2285–92. 10.3892/etm.2019.7795. PMC6676120 31410179

[16] Shi XB, Xue L, Yang J, Ma AH, Zhao J, Xu M, et al. An androgen-regulated miRNA suppresses Bak1 expression and induces androgen-independent growth of prostate cancer cells. Proc Natl Acad Sci U S A. 2007;104(50):19983–8. 10.1073/pnas.0706641104. PMC2148409 18056640

[17] Lydiatt WM, Patel SG, O’Sullivan B, Brandwein MS, Ridge JA, Migliacci JC, et al. Head and Neck cancers-major changes in the American Joint Committee on cancer eighth edition cancer staging manual. CA Cancer J Clin. 2017;67(2):122–37. 10.3322/caac.21389. 28128848

[18] Wang Y, Guo W, He Y, Chen Z, Wen D, Zhang X, et al. Association of MTHFR C677T and SHMT(1) C1420T with susceptibility to ESCC and GCA in a high incident region of Northern China. Cancer Causes Control. 2007;18(2):143–52. 10.1007/s10552-006-0097-4. 17206530

[19] Livak KJ, Schmittgen TD. Analysis of relative gene expression data using real-time quantitative PCR and the 2(-Delta Delta C(T)) Method. Methods. 2001;25(4):402–8. 10.1006/meth.2001.1262. 11846609

[20] Li J, Davidson D, Martins Souza C, Zhong MC, Wu N, Park M, et al. Loss of PTPN12 stimulates progression of ErbB2-dependent breast cancer by enhancing cell survival, migration, and epithelial-to-mesenchymal transition. Mol Cell Biol. 2015;35(23):4069–82. 10.1128/MCB.00741-15. PMC4628068 26391955

[21] Nair A, Chung HC, Sun T, Tyagi S, Dobrolecki LE, Dominguez-Vidana R, et al. Combinatorial inhibition of PTPN12-regulated receptors leads to a broadly effective therapeutic strategy in triple-negative breast cancer. Nat Med. 2018;24(4):505–11. 10.1038/nm.4507. PMC6675908 29578538

[22] Cao X, Chen YZ, Luo RZ, Zhang L, Zhang SL, Zeng J, et al. Tyrosine-protein phosphatase non-receptor type 12 expression is a good prognostic factor in resectable non-small cell lung cancer. Oncotarget. 2015;6(13):11704–13. 10.18632/oncotarget.3588. PMC4484488 25868976

[23] Piao Y, Liu X, Lin Z, Jin Z, Jin X, Yuan K, et al. Decreased expression of protein tyrosine phosphatase non-receptor type 12 is involved in the proliferation and recurrence of bladder transitional cell carcinoma. Oncol Lett. 2015;10(3):1620–6. 10.3892/ol.2015.3454. PMC4533285 26622721

[24] Wang X, Zhao X, Gao P, Wu M. c-Myc modulates microRNA processing via the transcriptional regulation of Drosha. Sci Rep. 2013;3:1942. 10.1038/srep01942. PMC3672885 23735886

[25] Li L, Yuan L, Luo J, Gao J, Guo J, Xie X. MiR-34a inhibits proliferation and migration of breast cancer through down-regulation of Bcl-2 and SIRT1. Clin Exp Med. 2013;13(2):109–17. 10.1007/s10238-012-0186-5. 22623155

[26] Rupaimoole R, Slack FJ. MicroRNA therapeutics: towards a new era for the management of cancer and other diseases. Nat Rev Drug Discov. 2017;16(3):203–22. 10.1038/nrd.2016.246. 28209991

[27] Gregory PA, Bracken CP, Smith E, Bert AG, Wright JA, Roslan S, et al. An autocrine TGF-beta/ZEB/miR-200 signaling network regulates establishment and maintenance of epithelial-mesenchymal transition. Mol Biol Cell. 2011;22(10):1686–98. 10.1091/mbc.E11-02-0103. PMC3093321 21411626

[28] Li Q, Zhang D, Wang Y, Sun P, Hou X, Larner J, et al. MiR-21/Smad 7 signaling determines TGF-β1-induced CAF formation. Sci Rep. 2013;3:2038. 10.1038/srep02038. PMC3687228 23784029

[29] Tili E, Michaille JJ, Wernicke D, Alder H, Costinean S, Volinia S, et al. Mutator activity induced by microRNA-155 (miR-155) links inflammation and cancer. Proc Natl Acad Sci U S A. 2011;108(12):4908–13. 10.1073/pnas.1101795108. PMC3064319 21383199

[30] Yao S, Xu J, Zhao K, Song P, Yan Q, Fan W, et al. Down-regulation of HPGD by miR-146b-3p promotes cervical cancer cell proliferation, migration and anchorage-independent growth through activation of STAT3 and AKT pathways. Cell Death Dis. 2018;9(11):1055. 10.1038/s41419-018-1059-y. PMC6192999 30333561

[31] Han PA, Kim HS, Cho S, Fazeli R, Najafian A, Khawaja H, et al. Association of BRAF V600E mutation and Micro RNA expression with central lymph node metastases in papillary thyroid cancer: a prospective study from four endocrine surgery centers. Thyroid. 2016;26(4):532–42. 10.1089/thy.2015.0378. PMC4827320 26950846

[32] Roskoski R Jr. The ErbB/HER family of protein-tyrosine kinases and cancer. Pharmacol Res. 2014;79:34–74. 10.1016/j.phrs.2013.11.002. 24269963

[33] Seshacharyulu P, Ponnusamy MP, Haridas D, Jain M, Ganti AK, Batra SK. Targeting the EGFR signaling pathway in cancer therapy. Expert Opin Ther Targets. 2012;16(1):15–31. 10.1517/14728222.2011.648617. PMC3291787 22239438

[34] Wu SG, Shih JY. Management of acquired resistance to EGFR TKI-targeted therapy in advanced non-small cell lung cancer. Mol Cancer. 2018;17(1):38. 10.1186/s12943-018-0777-1. PMC5817870 29455650

[35] Nair S, Bonner JA, Bredel M. EGFR Mutations in Head and Neck Squamous Cell Carcinoma. Int J Mol Sci. 2022;23(7):3818. 10.3390/ijms23073818. PMC8999014 35409179

[36] Zhou T, Chen T, Lai B, Zhang W, Luo X, Xia D, et al. FBXW2 inhibits prostate cancer proliferation and metastasis via promoting EGFR ubiquitylation and degradation. Cell Mol Life Sci. 2022;79(5):268. 10.1007/s00018-022-04320-3. PMC9061686 35499593

[37] Holbro T, Civenni G, Hynes NE. The ErbB receptors and their role in cancer progression. Exp Cell Res. 2003;284:99–110. 10.1016/S0014-4827(02)00099-X. 12648469

[38] Arab A, Yazdian-Robati R, Behravan J. HER2-Positive Breast Cancer Immunotherapy: A Focus on Vaccine Development. Arch Immunol Ther Exp (Warsz). 2020;68(1):2. 10.1007/s00005-019-00566-1. PMC7223380 31915932

[39] Terlikowska KM, Dobrzycka B, Terlikowski SJ. Chimeric Antigen Receptor Design and Efficacy in Ovarian Cancer Treatment. Int J Mol Sci. 2021;22(7):3495. 10.3390/ijms22073495. PMC8037934 33800608

[40] Miller DR, Ingersoll MA, Lin MF. ErbB-2 signaling in advanced prostate cancer progression and potential therapy. Endocr Relat Cancer. 2019;26(4):R195–209. 10.1530/ERC-19-0009. PMC6628717 31294537

